# NF-Y Dependent Epigenetic Modifications Discriminate between Proliferating and Postmitotic Tissue

**DOI:** 10.1371/journal.pone.0002047

**Published:** 2008-04-23

**Authors:** Aymone Gurtner, Paola Fuschi, Fiorenza Magi, Claudia Colussi, Carlo Gaetano, Matthias Dobbelstein, Ada Sacchi, Giulia Piaggio

**Affiliations:** 1 Molecular Oncogenesis Laboratory, Experimental Oncology Department, Regina Elena Cancer Institute, Rome, Italy; 2 Laboratory of Vascular Pathology, Istituto Dermopatico dell'Immacolata, Istituto di Ricovero e Cura a Carattere Scientifico (IRCCS), Rome, Italy; 3 Department of Molecular Oncology, Göttingen Center of Molecular Biosciences (GZMB), Göttingen, Germany; 4 Rome Oncogenomic Center, Experimental Oncology Department, Regina Elena Cancer Institute, Rome, Italy; Institute of Genetics and Molecular and Cellular Biology, France

## Abstract

The regulation of gene transcription requires posttranslational modifications of histones that, in concert with chromatin remodeling factors, shape the structure of chromatin. It is currently under intense investigation how this structure is modulated, in particular in the context of proliferation and differentiation. Compelling evidence suggests that the transcription factor NF-Y acts as a master regulator of cell cycle progression, activating the transcription of many cell cycle regulatory genes. However, the underlying molecular mechanisms are not yet completely understood. Here we show that NF-Y exerts its effect on transcription through the modulation of the histone “code”. NF-Y colocalizes with nascent RNA, while RNA polymerase II is I phosphorylated on serine 2 of the YSPTSPS repeats within its carboxyterminal domain and histones are carrying modifications that represent activation signals of gene expression (H3K9ac and PAN-H4ac). Comparing postmitotic muscle tissue from normal mice and proliferating muscles from mdx mice, we demonstrate by chromatin immunoprecipitation (ChIP) that NF-Y DNA binding activity correlates with the accumulation of acetylated histones H3 and H4 on promoters of key cell cycle regulatory genes, and with their active transcription. Accordingly, p300 is recruited onto the chromatin of NF-Y target genes in a NF-Y-dependent manner, as demonstrated by Re-ChIP. Conversely, the loss of NF-Y binding correlates with a decrease of acetylated histones, the recruitment of HDAC1, and a repressed heterochromatic state with enrichment of histones carrying modifications known to mediate silencing of gene expression (H3K9me3, H3K27me2 and H4K20me3). As a consequence, NF-Y target genes are downregulated in this context. In conclusion, our data indicate a role of NF-Y in modulating the structure and transcriptional competence of chromatin *in vivo* and support a model in which NF-Y-dependent histone “code” changes contribute to the proper discrimination between proliferating and postmitotic cells *in vivo* and *in vitro*.

## Introduction

A major question in biology today is how the chromatin environments are interrelated with the machinery that drives transcription, and how this impacts on biological processes. It is well known that the covalent modifications in the N-terminal tails of histones represent a key mechanism of transcriptional control in all eukaryotic cells, and that the functional link between chromatin structure and transcriptional activation is determined by the histone “code”. Two major players of this code consist in acetylation and methylation of specific lysine residues within histones H3 and H4 [1]–[4]. Generally, hyperacetylation correlates with transcriptional activation, while methylation of specific lysines is associated either with transcriptional repression or with activation [1], [5]–[7]. The enzymes that catalyze these transitions are histone acetyltransferases (HATs), histones deacetylases (HDACs) and histone methyltransferases (HMTs), respectively [8]. It seems clear that transcription factors, chromatin remodellers and histone modification enzymes work together to guide the cell through cell cycle and differentiation as well as cell transformation [1], [9]–[12]. Many transcriptional co-activators and co-repressor complexes with histone-modifying activities bind transcription factors *in vivo*, suggesting that targeted chromatin remodeling may be important for the function of transcription factors [13], [14]. Moreover, transcription factors like Myc, E2F, p53 and Rb are implicated in chromatin remodeling [15]–[20].

Based on these evidences, we investigated whether the transcription factor NF-Y exerts its activity as a master regulator of cell cycle progression, actively regulating the histone “code”. The histone-like transcription factor NF-Y is composed of three highly conserved subunits, NF-YA, -YB, and -YC, all required for binding of target sequences (CCAAT-boxes). NF-YA contains a transactivation domain at the N-terminus, whereas its DNA binding domain and the regions of interaction with NF-YB and NF-YC are located at the C-terminal portion of the protein [22], [23]. NF-Y supports basal transcription of several cell cycle regulatory genes, e. g. *E2F1*, *cyclin A*, *cyclin B1*, *cyclin B2*, *cdk1*, *cdc25A*, *cdc25C*, *chk2*, *dhfr*, *topoisomerase IIalpha*, and *mdr-1*, during different phases of the cell cycle and in response to DNA damage [16], [24]–[34]. In agreement with this, recent *in silico* studies point to NF-Y as a common transcription factor for an increasing number of cell cycle control genes [35], [36]. These findings strongly support the concept of the NF-Y complex as a key player in the regulation of proliferation and viability. It has been reported that the expression of a dominant negative NF-Y mutant blocks cell cycle progression in G1 and G2 [26], [37]. Most importantly, the knock out of the NF-YA subunit in mice leads to embryo lethality [38]. Recently, it has been suggested that NF-Y may modulate transcription via histone acetylation because of its interaction with the histone acetyl transferases p300/CBP and GCN5/PCAF [16], [27], [39]–[42]. In reporter gene assays, p300 enhances NF-Y-dependent transcription [43]. ChIP experiments have shown that NF-Y and p300 are dynamically bound to target promoters in the different phases of the cell cycle [16], [44]. However, it is not known whether NF-Y directly recruits HATs to chromatin, whether NF-Y leads to chromatin remodeling of its target promoters, and whether NF-Y acts upstream rather than downstream of histone tail modifications.

The data presented here clearly support the model of a direct role for NF-Y in chromatin remodeling *in vivo*. NF-Y binding leads to local histone hyperacetylation and correlates with hypo-methylation, while NF-Y target genes are actively transcribed. Local histone acetylation correlates with the recruitment of p300 and NF-Y. Loss of NF-Y binding, as induced by overexpressing a dominant-negative NF-Y protein, inhibits the activity of its target promoters and leads to local enrichment of methylated histones. Most notably, in terminally differentiated cells and muscle tissue, where NF-Y DNA binding is absent [45], NF-Y target genes exhibit a repressed heterochromatic state with the typical enrichment of H3K9me3, H4K20me3 and HP1α, and their expression levels are reduced. By contrast, in muscles from mdx mice, a murine model of human Duchenne muscular dystrophy with high rate of proliferating cells, NF-Y is expressed and binds its target promoters. In this system, NF-Y target genes are actively transcribed, and their promoters have an open chromatin structure. In conclusion, our data demonstrate that NF-Y regulates epigenetic modifications, and the NF-Y-dependent histone “code” changes contribute to the discrimination between a proliferative and a postmitotic status of the cells *in vivo*.

## Results

### NF-Y co-localizes with nascent mRNA and a transcription-permissive histone “code”

**Figure 1:**
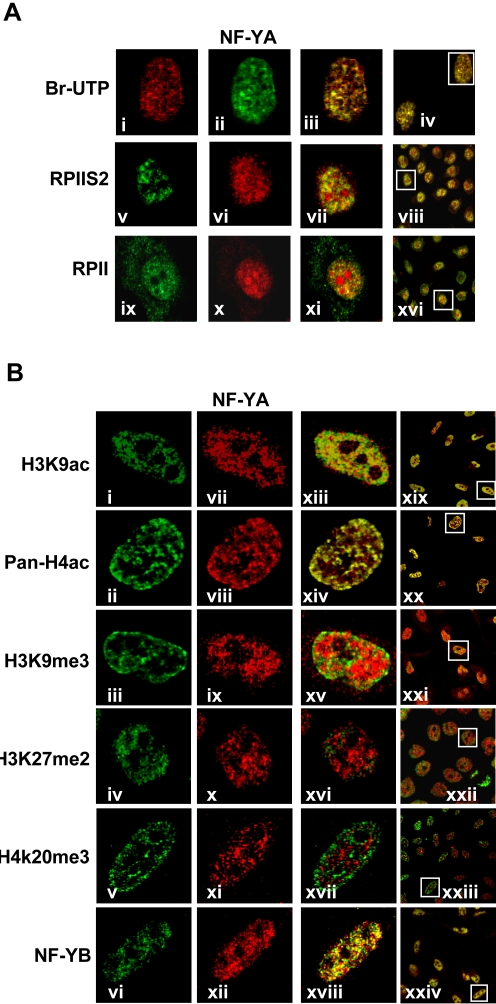
(A) NF-Y is associated with active transcription sites in living cells. After *in vivo* incorporation of BrUTP (run-on), cells were fixed and endogenous NF-YA (ii) and nascent RNA transcripts (i) were detected by indirect immunofluorescence combined with Confocal Scanning Laser Microscopy by using anti-NF-YA and anti-BrU antibodies. In the overlay (iii), yellow indicates colocalizations between NF-YA (green) and transcription sites (red). In panels vi and x cells were immunostained with anti-NF-YA, in panels v and ix with anti-RPII CTD repeat YSPTSPS (phospho S2) and anti-total RPII respectively. The majority of NF-YA (red) colocalizes with the activated form of RPII (green)(vii). (B) Cells were immunostained with anti-NF-YA (vii-xii), -acetylated H3K9 (i), -acetylated H4 (ii), -tri-methylated H3K9 (iii), -di-methylated H3K27 (iv), -tri-methylated H4K20 (v) and -NF-YB (vi) antibodies. The majority of NF-YA colocalizes with acetylated (xiii, xiv), but poor colocalization occurs with methylated histones (xv, xvi,xvii). Panel xviii shows the overlay of two subunits of NF-Y, NF-YA (xii) and NF-YB (vi). Panels from xix to xxiv represent a typical optical field of the merge. In figure 1A and 1B confocal analysis of single optical section is shown. The images have been collected with a 60x objective.

The involvement of NF-Y in transcription has been thoroughly reported. In agreement with this, we show in Figure 1A that in the nuclei of living cells, the NF-Y transcription factor colocalizes with nascent RNA Polymerase II (RPII) transcripts. We used a technique based on the incorporation of BrUTP into nascent RNA predominantly transcribed by RPII in living cells (run-on). Proliferating HeLa cells were transfected with BrUTP for 5′ at 4°C and then incubated for 5′ at 37°C; in this condition, most of the BrUTP-labeled RNA is still nascent [46]. After fixation, nascent RNA and endogenous NF-Y were visualized in a double-labeling experiment by Confocal Laser Scanning Microscopy, using antibodies against BrUTP and the NF-YA subunit, respectively. The anti BrUTP signal exhibited the typical distribution pattern of small spots which predominantly represent regions transcribed by RPII [46] (Figure 1A, panel i). Of note, a large amount of NF-YA (panel ii) colocalizes with nascent RNA (panel iii). Similar results have been obtained with an antibody against the NF-YB subunit (data not shown). To investigate whether NF-Y colocalizes with transcriptionally active RPII complexes, we used an antibody directed against the RPII protein phosphorylated at the serine 2 residue of the consensus repeat (Y1S2P3T4S5P6S7) contained in its carboxyterminal domain (CTD), the transcriptionally active form of RPII [47] (Figure 1A, panel v). In agreement with the results of the run-on, we found a significant colocalization (panel vii) between RPIIS2 (panel v) and NF-YA (panel vi). Since it is known that this phosphorylation occurs during transcriptional elongation, these results, together with the localization of NF-YA at nascent RNA , strongly support the notion that NF-Y is present at active transcription foci. As a control, we also show the colocalization (panel xi) of NF-YA (panel x) with total RPII (panel ix). Next, we asked whether NF-Y colocalizes with post-translational modifications on histones that are known as positive marks of transcription. Figure 1B shows that the majority of NF-YA (panels vii-xii) colocalizes (panels xiii, xiv) with the transcription permissive histone modifications H3K9ac and PAN-H4ac (panel i and ii), while poor colocalization is observed with marks of close chromatin conformation, H3K27me2 (panel iv) and H4K20me3 (panel v). NF-YA weakly colocalized (panel xv) with H3K9me3 (panel xv). As a positive control, the colocalization of NF-YA and -YB subunits is shown (Figure 1B, panel xviii). Interestingly, we observed that NF-YA could also localized in to the nucleoli. This result is in agreement with our previous observations with endogenous and exogenous overexpressed NF-YA (G.P. personal observation). The amount of NF-YA in the nucleoli is variable, being more detectable in some cells (Figure 1A, panels vi and x) then in others (Figure 1B, panels xiii-xviii). We never observed a completed exclusion of this protein from the nucleoli, thus it will be interest in the future investigate which could be the meaning of NF-YA nucleolar localization. Taken together, these results indicate a direct interaction between NF-Y and actively transcribed chromatin regions with an open chromatin structure.

### NF-Y binding correlates with a euchromatic conformation of its target promoters

**Figure 2:**
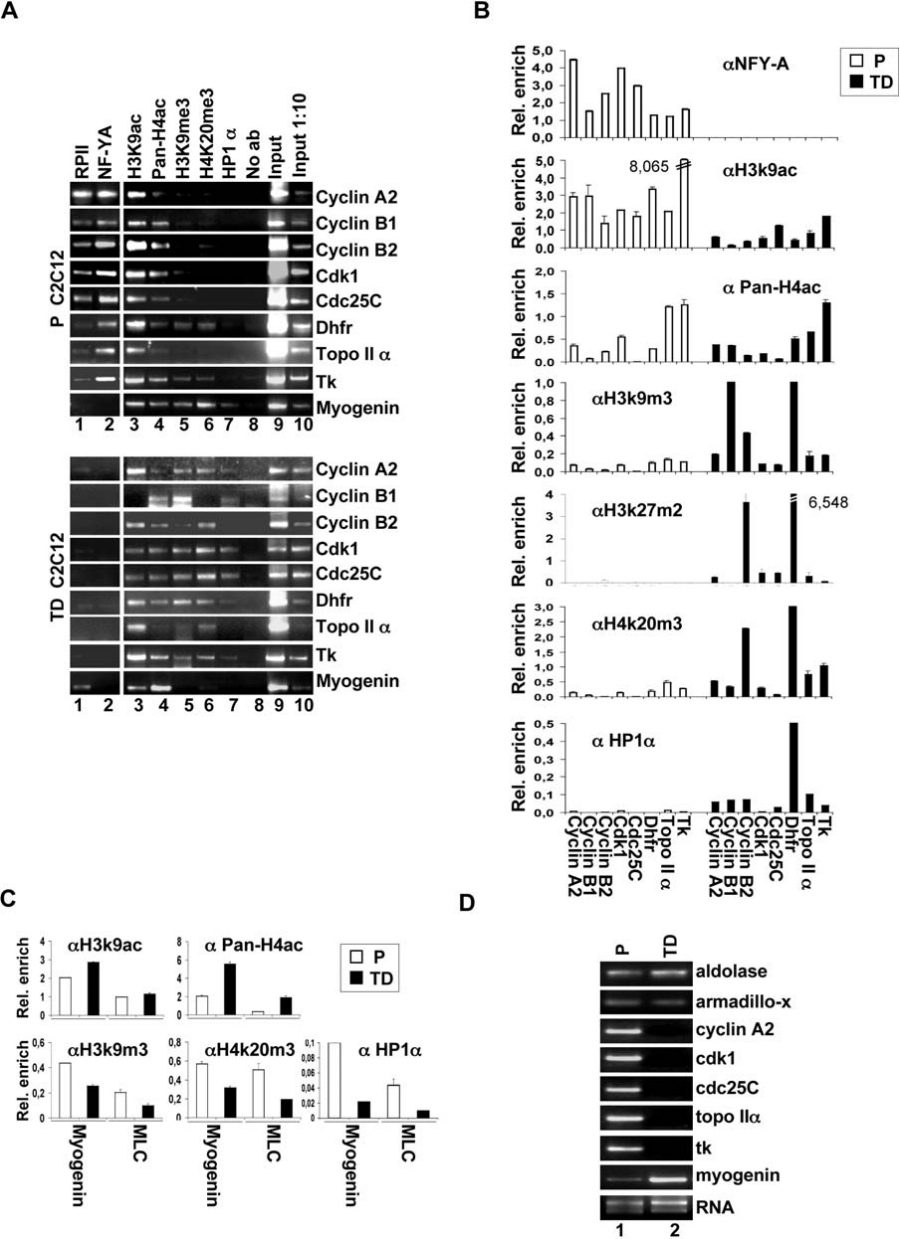
Histone acetylation correlates with NF-Y recruitment onto its target promoters. (A) ChIPs performed on proliferating (P) and terminally differentiated (TD) C2C12 cells using the indicated antibodies. No antibody was used as control (No Ab). (B) Q-ChIP analysis on proliferating (P) and terminally differentiated (TD) primary myoblasts, performed with the indicated antibodies. The promoters analyzed were: 1) *CyclinA2,* 2) *CyclinB1*, 3) *CyclinB2,* 4) *Cdk1*, 5) *Cdc25C,* 6) *Dhfr,* 7) *TopoisomeraseIIα,* 8) *Tk.* (C) Q-ChIP analysis of the *Myogenin* and *MLC* promoters performed with the indicated antibodies on proliferating (P) and terminally differentiated (TD) primary myoblasts. On B and C the enrichment of immunoprecipitated promoter fragments relative to the input was done. Rabbit IgG was used as control. (D) RT-PCR amplification of indicated genes was performed on proliferating (P) and terminal differentiated (TD) C2C12.

In order to study the role of NF-Y binding activity in histone modifications at multiple genomic loci, we performed ChIP experiments in proliferating (P) and terminally differentiated (TD) C2C12 skeletal muscle cells. This cells line is a very useful physiological system to study the relationship between NF-Y and the mitotic status. Indeed, when C2C12 cells are actively proliferating, NF-Y serves as a common transcription factor for several key cell cycle control genes, and when cells are induced to differentiate in myotubes, upon growth factors withdrawal, a permanent down regulation of NFY-A expression leads to the inhibition of NF-Y binding to its target promoters [45]. Consistent with these findings, ChIP analysis demonstrates that in P cells, NF-Y is recruited onto all tested promoters (*Cyclin A2*, *Cyclin B1*, *Cyclin B2, Cdk1, Cdc25C, Dhfr, Topoisimerase II α, Tk)* (Figure 2A, lane 2). This binding is associated with the presence of RPII (Figure 2A, upper panel, lane 1) and the transcription permissive histone marks H3K9ac and PAN-H4ac (lanes 3 and 4). Moreover, the chromatin of NF-Y target promoters is characterized by low levels of the heterochromatin marks H3K9me3 and H4K20me3 (lanes 5 and 6) [48], and the heterochromatin protein 1 (HP1α) (lane 7). In contrast, in TD cells, the lack of NF-Y binding (Figure 2A, lower panel, lane 2) correlates with the lack of RPII binding (lane 1) and the induction of H3K9me3 and H4K20me3 (lanes 5 and 6). The presence of methylated histones is accompanied by the recruitment of heterochromatin protein 1 (HP1) (lane 7), a chromodomain protein that specifically recognizes H3K9me3 [49] and is thought to be involved in organizing a higher-order chromatin structure [48]. In agreement with this, NF-Y target cell cycle regulatory genes are not transcribed in TD cells (Figure 2D, lane 2). We did not observe a substantial reduction in the association of PAN-H4ac and H3K9ac with NF-Y target promoters in TD cells. The pattern of acetylated and methylated histones is different when we analyze the *Myogenin* promoter (Figure 2A, upper and lower panel). This promoter is not bound by NF-Y (Figure 2A, lane 2) and is actively transcribed in TD cells (Figure 2D, lane 2).

We extend these observations to primary myoblasts, which are the proliferating progenitors of myotubes. These cells were obtained from skeletal muscles of newborn mice and maintained in culture in the presence of high serum concentration and basic fibroblast growth factor (bFGF) to promote proliferation and prevent differentiation. Muscle differentiation was induced by serum withdrawal [50]. In this cell system, we performed ChIP experiments followed by Q-PCR analysis (Q-ChIP). Also in primary myoblasts, as well as in P C2C12 cells, the transcription permissive histone marks H3K9ac and PAN-H4ac are associated with NF-Y target promoters (Figure 2, panel B). Again, this chromatin is characterized by low levels of the heterochromatin marks H3K9me3, H4K20me3, H3K27me2, and pHP1α. In contrast, in myotubes, the chromatin of NF-Y target promoters strongly associates with H3K9me3, H4K20me3, H3K27me2 and pHP1α . Although we did not observe a substantial reduction in the association of PAN-H4ac with NF-Y target promoters in myotubes, the association of H3K9ac is strongly reduced. The pattern of acetylated and methylated histones is different if we analyze the promoters of two muscle genes that are not regulated by NF-Y, *Myogenin* and *Myosin Light Chain (MLC)* (Figure 2C). All together, these results suggest that, both in culture and in primary muscle cells, the association of NF-Y with CCAAT boxes correlates with hyperacetylation of histones H3 and H4; in contrast, the absence of NF-Y binding to promoters of cell cycle regulatory genes correlates with a local recruitment of heterochromatic markers, such as H3K9me3, H4K20me3.

### NF-Y recruits p300 onto its target promoters

**Figure 3:**
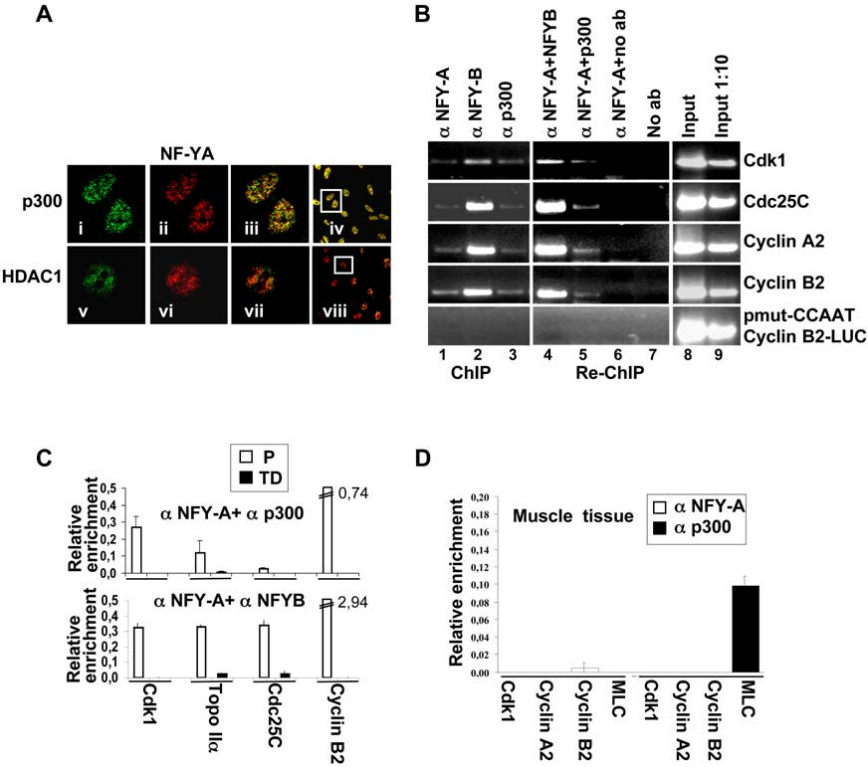
NF-Y recruits p300 onto its target promoters. (A) Cells were immunosteined with anti-NF-YA (ii, vii), anti-p300 (i), anti-HDAC1 (v) antibodies. Panels iii and vii show the overlay of two proteins, panels iv and viii represents a typical optical field of merge. Confocal analysis of single optical section is shown. (B) ChIPs and re-ChIPs experiments performed with anti-NF-YA, -NF-YB and –p300 antibodies, on proliferating C2C12 cells and stable transfected C2C12 with pmut-CCAATcyclinB2-LUC vector. PCR analysis were performed on the immunoprecipitated DNA samples using specific primers for the indicated promoters and specific primers for the exogenous cyclin B2 mutated promoter. No antibody was used as control (No Ab). (C) Q-Re-ChIP analysis performed on proliferating (P) and terminally differentiated (TD) C2C12 cells using the indicated antibodies on the sames promoters of panel B. (D) Q-ChIP experiments performed with anti-NF-YA, –p300 antibodies, using chromatin prepared from adductor muscles of wt (white bars) and mdx (black bars) of C57BL/10 mice. Specific primers for *Cdk1, Cyclin A2, Cyclin B2* and *MLC* promoters. The values represent the mean of five indipendent experiments performed on triplicate.

We and others, have previously reported that the HAT p300 enzyme binds NF-Y target promoters, and that its binding correlates with the presence of highly acetylated histones. Notably, HDAC1 recruitment is inversely correlated to that of p300, [16], [43], [44]. From the results obtained until now, we hypothesize that NF-Y might control the transcription of its target genes through local recruitment of histone-modifying enzymes. Therefore, we performed experiments to verify whether NF-Y might recruit the p300 enzyme onto its target promoter sequences and consequently allows specific recruitment of acetylated histones. First, we investigated the colocalization pattern of NF-Y and p300 or HDAC1 (Figure 3A).. As expected, NF-YA (panel ii) colocalizes (panel iii) with p300 (panel i), while poor colocalization (panel vii) is observed between NF-YA (panel vi) and HDAC1 (panel v).

Re-ChIP experiments offer an ideal means to test whether NF-Y and p300 are co-recruited onto CCAAT containing promoters. With the first ChIP, performed with antibodies against NF-YA, -YB and p300 proteins, we observed that, as expected, NF-Y and p300 are recruited on the same regions of *Cdk1, Cdc25C, Cyclin A,* and *Cyclin B2* promoters (Figure 3B, lane 1,2,3). After we performed Re-ChIP analysis, immunoprecipitating the NF-YA-containing complexes with an antibody against p300, only those DNA sequences that are simultaneously bound by both proteins would be amplified in the subsequent PCR. Our results show that p300 and NF-Y are co-recruited at the same time on the same DNA fragments (Figure 3B, lane 5) and suggest that the two factors co-occupy common target loci. As expected, NF-YA and -YB subunits together bound all tested promoters (lane 4). The p300 Re-ChIP signals were not due to a non specific background, since when the NF-YA-containing complexes were subjected to a second immunoprecipitation without antibody, no DNA was immunoprecipitated (lane 6).

To assess whether the presence of NF-Y is required for p300 recruitment, we performed ChIP experiments on a mixed population of cells stably transfected with a cyclin B2 promoter carrying mutated CCAAT-boxes, linked to a luciferase gene (pmut-CCAATCyclinB2-LUC). As expected, NF-Y does not bind the mutated consensus sequences, as previously reported [24], [49]. Of note, we observed that p300 is not recruited onto this DNA (Figure 3B, lane 3), and accordingly, no signals in Re-ChIP experiments were detected (Figure 3B, lanes 4 and 5). We quantify these results by Q-Re-ChIP analysis performed on P and TD C2C12 cells (Figure 3C). Although at different extent, we confirm that NF-Y and p300 are co-recruited on several NF-Y target promoters. As expected, no recruitment of p300 is observed in TD C2C12 cells, where NF-Y is not expressed. In agreement with this, by Q-ChIP analysis performed on murine muscle tissue (Figure 3D), we observed that the absence of NF-Y binding on its target promoters is associated with the absence of p300 recruitment. Notably, also in murine muscle tissue, as observed in different cell lines (16, 43, 44, AG unpublished data), HDAC1 recruitment is inversely correlated to that of p300. (Figure 5 D) The results shown here strongly support a direct role of NF-Y on the p300 recruitment to the NF-Y targets we have analyzed. Of note, the loss of p300 recruitment in the absence of NF-Y binding is a common feature of both cell cultures (C2C12) and murine muscle tissue, supporting its relevance in animals.

### Loss of NF-Y binding allows recruitment of methylated histones

**Figure 4:**
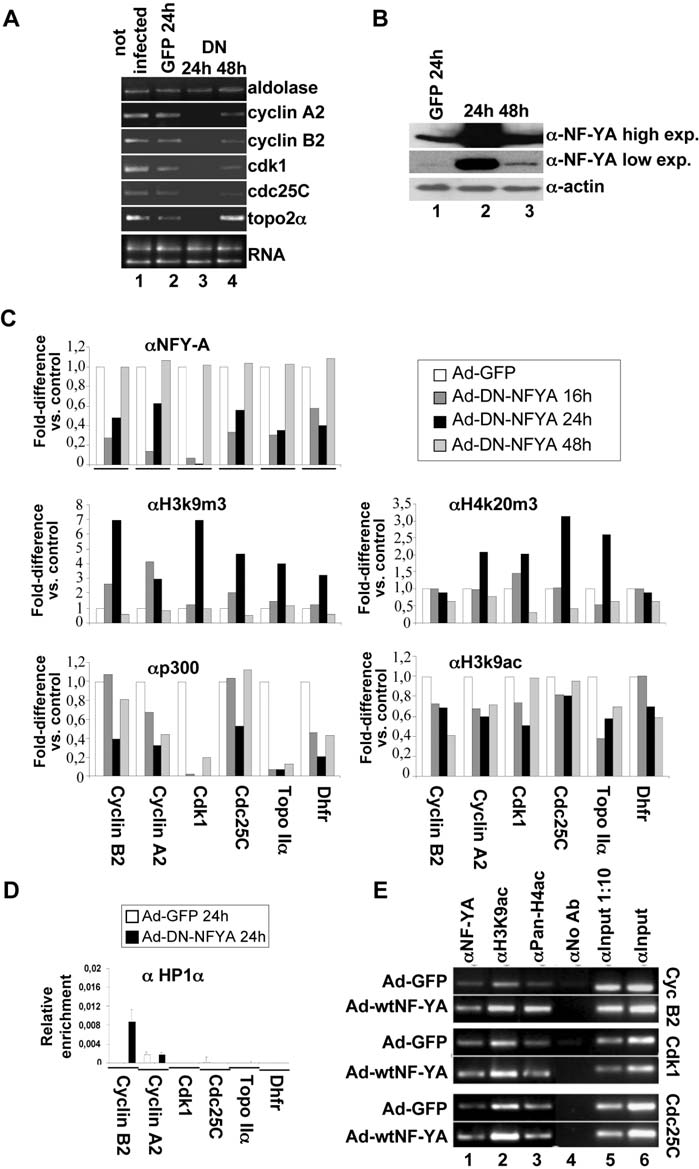
Lack of NF-Y DNA binding activity induces hypermethylation of histones and target gene inactivation. (A) RT-PCR amplification of the indicated genes from uninfected MEFs (lane 1), MEFs 24hours after infection with Ad-GFP (lane 2), MEFs 24hours and 48hours after infection with Ad-DN-NFYA (lanes 3 and 4). (B) Western blot analysis performed on total lysates from: Mef after 24hours post infection with Ad-GFP (lane1), Mef after 24 hours post infection with Ad-DN-NFYA (lane 2), Mef after 48 hours post infection with Ad-DN-NFYA (lane 3). The extracts were probed with rabbit polyclonal with anti-NFY-A. To normalised protein's loading, the filter was stained with a monoclonal antibody anti-actin protein. (C) Q-ChIP analysis, of the indicated NF-Y target promoters on MEF after 16, 24, 48 hours (h) from Ad-GFP (white bars), or Ad-DN-NFYA after 16 h from infection (dark grey bars), 24 h (black bars), 48 h (grey bars), performed with anti-NFY-A, -p300, -H4K20me3, -H3K9me3 and -H3K9ac antibodies. The fold difference value in each case compares the sample performed after Ad-DN-NFYA infection to the corresponding control sample performed after Ad-GFP infection at the same time point (defined as 1). One of two independent experiments performed in triplicate is represented. (D) Q-ChIP analysis, performed with anti-HP1α antibody, of the indicated NF-Y target promoters on MEF after 24 h after infection with Ad-GFP (white bars) and Ad-DN-NFYA (black bars). It is shown one of two independent experiments performed in triplicate. (E) ChIPs were performed on proliferating MEFs 24 hours after Ad-GFP and wtNF-YA infections. No antibody was used as control (No Ab). PCR analysis were performed on the immunoprecipitated DNA samples with anti-NFY-A, -H3K9ac and -PAN-H4ac-Pan antibodies, using specific primers for the indicate promoters.

In order to further address the direct role of NF-Y in chromatin remodeling, we overexpressed in mouse embryo fibroblasts a dominant negative mutant form of NF-Y (DN-NF-YA). This molecule is a NF-YA protein with a triple amino acid substitution in the DNA binding domain that impairs its ability to bind DNA. It is still able to interact with a NF-YB/-YC dimer, but the resulting trimer is inactive in terms of CCAAT recognition [52]. First, we investigated whether DN-NF-YA affects the expression of NF-Y target genes. RT-PCR analysis performed 24 hours post infection with an adenovirus vector expressing DN-NF-YA (Ad-DN-NF-YA) shows that cyclin A2, B2, cdk1, cdc25C, topoisomerase IIα mRNAs were greatly down-regulated (Figure 4A, lane 3). The cell cycle gene expression is only transiently repressed in the presence of DN-NF-YA, as indicated by RT-PCR analysis performed 48 hours after infection (Figure 4A, lane 4). The transient effect observed might be attributed to the short half-life of the mutant protein whose expression 48 hours after infection is strongly reduced (Figure 4B, lane 3). Next, we investigated the DNA recruitment of NF-Y, p300 and different histone post-translational modifications after transduction with the adenovirus vector expressing DN-NF-YA . Q-Chip analysis shows that, although to different extents, the overexpression of DN-NF-YA leads to a decrease in the recruitment of NF-Y to *Cyclin B2, A2, Cdk1, Cdc25C, Topoisomerase IIα,* and *Dhfr* promoters at 16 and 24 hours post transduction (Figure 4C). Of note, the decrease of NF-Y DNA binding activity caused a strong increase of H3K9me3 and H4K20me3, with a peak at 24 hours, while it caused only a partial reduction of H3K9ac and p300 (Figure 4C). In good agreement with the short half-life of the mutant NF-YA, the NF-Y binding activity is restored 48 hours post infection. Concomitantly we observe a strong decrease of methylated histones accompained by a partial recovering of the p300 binding and H3K9ac levels. Thus, the most significant event induced by loss of NF-Y binding is an increased recruitment of closed chromatin markers, and not a decrease of open chromatin markers. The extent of the effect of the DN-NF-YA, both on NF-Y and chromatin marks binding, is variable on the different tested promoters. This might depend on the different dynamic with which the wt NF-Y trimer is replaced by the ones carrying the mutant NF-YA protein, in the context of the different chromatin structures of the promoters. However, the biological effect in terms of chromatin structure and transcription is very similar. Interestingly, the results of the Q-ChIP experiments performed after infection with the DN-NF-YA expression vector indicate that the heterochromatin protein HP1α does not bind these promoters although a week recruitment is observed on cyclin B2 promoter (Figure 4D).

The consequences of NF-YA overexpression were also evaluated. For this purpose, we performed ChIP experiments in cells infected with an adenovirus carrying the wild type NF-YA subunit (Ad-wtNF-YA), that acts as the regulatory subunit of the trimer [24]. The overexpression of NF-YA leads to a detectable increase of NF-Y binding to its target promoters (Figure 4E, lane 1). This increase was sufficient to induce hyperacetylation of histones H3 and H4 on *Cyclin B2*, *Cdk1* and *Cdc25C* promoters (Figure 4E, lanes 2 and 3). Taken together, our data demonstrate that NF-Y DNA binding activity is essential for transcription of its target genes, and it correlates with accumulation of acetylated histones. Moreover, lack of NF-Y binding results in lack of transcription and correlates with the accumulation of H3K9me3 and H4K20me3 histones on its target promoters.

### Recruitment of euchromatic marks correlates with NF-Y binding in adult tissues

**Figure 5:**
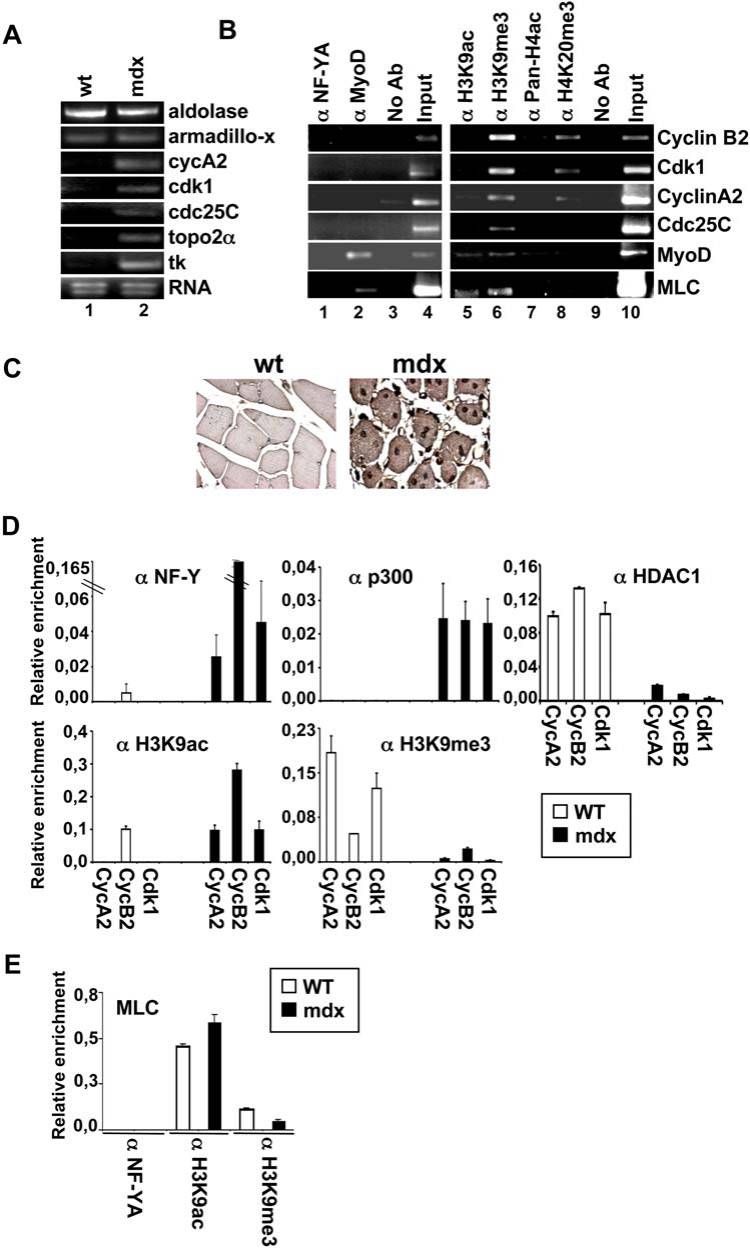
Recruitment of euchromatin marks correlates with NF-Y binding in adult tissues. (A) RT-PCR amplifications of indicate genes were performed on wt and mdx murine muscle total RNAs. Aldolase and Armadillo-X-linked gene were used to normalize. (B) ChIPs performed on wt SDF1 murine muscle tissue using the indicated antibodies. PCR were performed using specific primers for the indicated promoters. (C) Immunohystochemical analysis of wt and mdx muscle tissue using an antibody specific for NF-YA. (D) Q-ChIP analisys of the indicate NF-Y target promoters, performed on muscles of wt (white bars) and mdx (black bars) of C57BL/10 mice, using the indicated antibodies. (E) Q-ChIP analysis of Myosin Light Chain (MLC) muscle specific gene, performed on muscles of wt (white bars) and mdx (black bars) of C57BL/10 mice. The results shown in D and E are the mean of five independent experiments performed in triplicate.

To study the chromatin remodeling of NF-Y target promoters in a physiological model system, we set up the ChIP technique on murine muscle tissue. As assessed by RT-PCR experiments, in this system, NF-Y target transcripts are downregulated (Figure 5A, lane 1), as expected for a terminally differentiated tissue where the majority of the cells should be arrested in a postmitotic status. In good agreement with the results reported above for terminally differentiated C2C12 cells, Figure 5B (lane 1) shows that in the absence of NF-Y binding to its target promoters a specific induction of methylated histones (H3K9me3 and H4K20me3) is detectable (lanes 6 and 8, respectively), which corresponds to the absence of the acetylated ones (H3K9ac and PAN-H4ac) (lanes 5 and 7, respectively). Thus, adult muscle tissues are characterized by loss of NF-Y binding , concomitant accumulation of negative and reduction of permissive marks of transcription. These features concur to the maintenance an irreversible close chromatin conformation of NF-Y target genes. The pattern of acetylated and methylated histones is different if we analyze promoters that are not regulated by NF-Y and are actively transcribed in muscle tissues as *MyoD* and *Myosin Light Chain* (*MLC*). As expected, MyoD is recruited on these promoters *in vivo* (Figure 5B, lane 2). Interestingly, in this case, it is evident that acetylated histone H3K9ac is strongly increased (lane 5), confirming an open chromatin structure of these promoters, although H3K9me3 is still present (lane 6). The concomitant presence of acetylated and methylated histones H3K9 is not of obvious explanation, since histone H3K9 cannot be simultaneously acetylated and methylated within an individual histone amino tail. It might be speculated that different histone amino tail molecules are either acetylated or methylated, and that the balance between these two signals will result in the appropriate chromatin configuration. Indeed, it has been recently demonstrated that the distribution of these two histone marks on the promoter regions of transcriptionally active genes overlap each other (53). The simultaneous presence of acetylated and tri-methylated H3K9 in the same chromatin domain might antagonize the silencing activity normally associated with H3K9 methylation.

We tested the ability of NF-Y binding in modulating the recruitment of different modified histones onto its target DNA also in muscle of *mdx* mice that represent an animal model for human Duchenne and Becker muscular dystrophy. The muscle of mdx mice displays a high regenerative capacity leading to muscle hypertrophy. Interestingly, in these mice, a degeneration and necrosis of individual fibers is accompanied by proliferation of the satellite cells [54], providing an attractive model to investigate the role of NF-Y in chromatin remodeling events *in vivo*. First, we observed that NF-YA is expressed in the nuclei of the mdx muscle fibers, whereas it is absent in wt muscle nuclei (Figure 5C). Consistent with this observation, RT-PCR analysis indicate that NF-Y target transcripts are expressed on mdx muscle tissues (Figure 5A, lane 2). By Q-ChIP analysis (Figure 5D), performed on five different wt and mdx mice, we found that NF-Y binds the promoters of these genes in mdx, and that this binding is associated with the recruitment of p300, an induction of H3K9ac and a lack of H3K9me3 on the same promoter regions We have previously shown that on NF-Y target promoters HDAC1 recruitment is inversely correlated to that of p300 [16]. In agreement with this, we observed a displacement of HDAC1 from NF-Y target promoters in mdx muscle. Loss of NF-Y binding in wt muscle is associated with the induction of methylated H3 in lysine9, the reduction of acetylation on the same lysine, the loss of p300 and the recruitment of HDAC1. Notably, we confirm the same results on two different wt mice strain (SDF1, C57BL/10). As expected, NF-Y doesn't bind *in vivo* the MLC promoter (Figure 5E). We observed a similar level of acetylated histones onto MLC promoter independently of the muscle tissues studied (wt and mdx). This shows the homogeneity of the cell types represented in the tissues, thus confirming our data of Q-ChIP experiments from muscle tissues.

Taken together, these results demonstrate that, also in adult tissues *in vivo*, the presence of NF-Y correlates with euchromatic markers and a mitotic status of cells. In contrast, the absence of this transcription factor is associated with the presence of heterochromatic markers and a postmitotic status of cells.

## Discussion

As cells progress through the cell cycle and differentiation, they must ensure that the right sections of their genomes are activated and repressed at the appropriate times. This is achieved mainly by regulating the dynamic association of DNA with histone and non-histone proteins, thus by regulating chromatin structure. The results presented in this work provide evidences that there is a spatial relationship between newly synthesized RNA transcription-permissive histone “code” and NF-Y *in vivo*. Consistent with this, NF-Y DNA binding activity modulates the chromatin structure of its target genes through the local maintenance of post-translationally modified histones, resulting in gene transcription modulation. Our ChIP data demonstrate, for the first time, that NF-Y binding to CCAAT boxes correlates with the presence of positive (H3K9ac and pan-H4ac) and the absence of negative (H3K9me3, H3K27me2, H4K20me3 and HP1α) transcription marks indicating that NF-Y binding to DNA could prevent hypermethylation of histone tails. By contrast, in the absence of NF-Y binding to promoter of cell cycle genes, there is a substantial local recruitment of heterochromatic markers, such as H3K9me3, H3K27me2, H4K20me3 and HP1, known to be signals of gene silencing [5], [19], [55]. Thus, we speculate here that NF-Y might influence the balance between euchromatin and heterochromatin, regulating active and negative histone modification marks on its target promoters.

The significance of these findings is strongly reinforced by the fact that we confirm our results not only in cultured and primary cells but also in adult mice muscle tissues. Furthermore, the biological relevance of our observations resides also in the fact that NF-Y controls the expression of a large amount of key cell cycle regulator genes [35], [36] . Conversely, upon differentiation, the suppression of NF-Y activity has been reported to be crucial for the inhibition of cell cycle regulatory gene expression and the induction of early differentiation markers [45]. Since NF-Y is ubiquitously expressed and the cell cycle regulatory genes are silenced in the majority of differentiated tissues, the molecular mechanism that we describe in muscle could represents a common pathway for cell cycle gene silencing during differentiation of several tissues.

**Figure 6:**
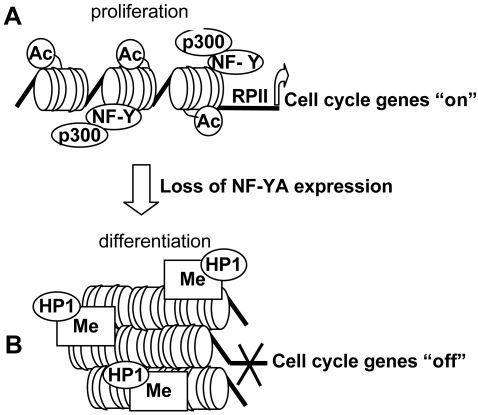
Model proposing the molecular mechanism underlying the transcriptional control of cell cycle-related genes by NF-Y.

Our results support a model (Figure 6) in which transcription factors, chromatin remodellers and histone modification enzymes work together to guide the cell through cell cycle and differentiation. According to this model, we have demonstrated that NF-Y DNA binding activity correlates with the recruitment of p300 and the maintenance of acetylated histones on its target promoters (Figure 6A). These results are in agreement with previous ones, indicating that NF-Y is required to establish a promoter architecture that facilitate transcription [41], the pattern of p300 binding closely matches that of NF-Y binding and promoter activation [44], and that p300 physically interacts with NF-Y [16], [Bibr pone.0002047-Huang1] [56 55].

Our results on murine muscle tissues strongly suggest a model in which NF-Y loss is required for heterochromatinization of cell cycle regulatory genes and consequently represents a common pathway for cell cycle gene silencing during differentiation (Figure 6B). It has been previously demonstrated that the HMT suv39h is required for myoblastic differentiation. In particular, this protein is essential for H3K9 methylation and subsequent repression of E2F target genes involved on G1/S transition, like *Cyclin E, Cyclin D,* and *Dhfr* [10]. In the present paper we demonstrate that, throughout muscle differentiation, also NF-Y target genes are silenced by epigenetic gene regulation. Indeed, the absence of NF-Y binding to promoters of cell cycle regulatory genes, involved in the S/G2 and G2/M transitions, is associated with the local enrichment of negative marks of transcription such as H3K9me3, HP1α and H4K20me3. The recruitment of the heterochromatic protein HP1α on NF-Y target promoters could explain how they are irreversibly locked in a repressed state. Our results obtained after overexpression of the DN-NF-YA and upon terminal differentiation *in vitro,* demonstrate that the major event induced by loss of NF-Y is an increase of histones methylation, rather than a decrease of acetylation, supporting the idea that NF-Y binding activity prevents the hypermethylation of histones, allowing the transcription of cell cycle genes. This could mean that the recruitment of methylated histones is sufficient to silence the transcription of cell cycle regulatory genes, inducing an arrest of proliferation. On the other hand, the data obtained on muscle tissues *in vivo* show that the loss of NF-Y activity leads also to a loss of acetylated histones. Therefore, these results let us to speculate that the chromatin state of cell cycle regulatory genes might be more permissive for transcription upon terminal differentiation *in vitro* rather than in tissues *in vivo*. In other words, in *in vitro* differentiation systems, cell cycle regulatory genes might be ‘primed’ for repression but ‘held in check’ by an opposing histone modification. This scenario resembles the recently described unusual ‘bivalent’ chromatin structure occurring at promoters of master genes in ES cells, where both active and repressive chromatin marks are physically present at the same nucleosomes [53], [57], [58].

In good agreement with our data, it has been reported that several vertebrate transcription factors involved in the cell cycle progression recruit active marks of transcription. For example, the recognition of Myc to its binding sites is based on specific epigenetic context and, once bound to DNA, Myc locally enhances histone acetylation [18], [59]. It has been shown that chromatin association of activating E2F-complexes is required to promote histone acetylation during the cell cycle [20]. Less is known about the role of these factors in recruiting negative marks of transcription to their target genes when the cells exit the cell cycle and reach a postmitotic status. However, it has been recently described that down-regulation of Myc expression leads to a widespread decrease of active and increase of repressive chromatin marks [60], suggesting that Myc might influence chromatin structure of its target promoters with the same molecular mechanism we describe here for NF-Y.

## Materials and Methods

### Cell culture, DNA transfection and infection, plasmids

Murine C2C12 myoblasts and HeLa cells were cultured in DMEM supplemented with 10% FBS. Murine embryo fibroblast (MEF) was cultured in DMEM supplemented 15% FBS. Primary myoblasts were explanted from new-born mice andmaintained in culture as described [50]. To avoid spontaneous differentiation, primary satellite and C2C12 cells were never allowed to reach confluence. Muscle differentiation was induced by plating the cells into collagen-coated dishes and switching them for 72 h to serum free (SF) medium. DMEM was supplemented with Redu-Ser (Upstate Biotechnology Incorporated, Lake Placid, NY) to a final concentration of 5 µg/ml human insulin, 5 µg/ml human (holo) transferrin, 5 ng/ml sodium selenite. 50 µM cytosine arabinoside (Ara-C) was added to the SF medium eliminate undifferentiated cells. For immunofluorescent labeling and run-on, the cells were seeded onto glass cover-slips, pretreated with L-lysine, mounted on the bottom of 35-mm petri dishes and grown for 24 hours in DMEM containing 10% FBS. Stable transfections were performed as described previously [25]. The Luc-reporter constructs used in transfection experiments was: pmutCCAAT-cyclinB2LUC [24]. Recombinant adenoviral vectors encoding DN-NF-YA, wtNF-YA and GFP have been described previously [27]. Mouse embryo fibroblasts were grown in DMEM containing 15% FBS and used at 3° passage. Cells exponentially growing on plates were infected in DMEM without serum for 1 hour at 37° C at a multiplicity of infection (MOI) of 400. Following infection DMEM supplemented with 15% FBS were added to each plate and the cells were incubate at 37°C and analyzed at the indicated time-point.

### Labeling of Nascent RNA (run-on)

Labeling of nascent RNA was carried out on intact living cells as previously described [46] with some modifications. Briefly, living cells were transfected with 3mM 5-bromouridine 5′-triphosphato (BrUTP) (Sigma-Aldrich s.r.l.) complex with the non-liposomal transfection reagent FuGENE™ 6 [61]. 10% of FuGENE 6 was diluted in HEPES 20mM-saline buffer pH7.4 and incubate at room temperature for 5 minutes. Than 3mM of BrUTP was added to mixture and incubate for 20 minutes at room temperature. 20–50 ml drops were transferred to cells. After 5 minutes at 4°C the coverslips were quickly washed in phosphate buffered saline (PBS) pre-warmed to 37°C and returned to their wells with 10% FBS medium for a pulse of 5 minutes at 37°C. Cells were quickly fixed for 10 minutes in 2% (w/v) formaldehyde. The BrUTP incorporated into nascent RNA was visualized by indirect immunofluorescent labeling and the run-on efficiency was calculated as 39% (data not shown). Control cultures treated with FuGENE 6 only and treated with 100 ug/ml DNase-free (heated at 95°C for 10 minutes) RNase for 30 minutes at room temperature followed by washing and further processing for immunofluorescence analysis, were included (data not shown).

### Double Immunofluorescence labeling

Cells were fixed for 10 minutes with 2% (w/v) formaldehyde in PBS. The following primary antibodies (diluted in 1% BSA) were used: anti-NF-YA rabbit polyclonal (on run on experiments) (Rockland n°200-401-100, Gilbertsville, PA), anti-NF-YA mouse monoclonal from hybridoma cells, anti-NF-YB rabbit polyclonal (gifts from R. Mantovani), anti-BrUTP (Roche Diagnostics n°1170376, clone BMC 9318), anti-RPII (Santa Cruz n° 899), anti- RPIIS2 (Abcam n°5095), anti-PAN-H4ac (Upstate n°06-598), anti-H3ac-k9 (Abcam n°4441), anti-H3tri-m-k9 (Abcam n°8898), anti-H4tri-m-k20 (Abcam n°9053), anti-p300 (Santa Cruz n° 584 and 585), anti-H3di-m-k27 (Abcam n°24684) and anti-HDAC1 (Sigma H3284). The following secondary antibodies (diluted in 1% BSA) were used: Cy3-conjugated donkey anti-mouse and Cy2-conjugated donkey anti-rabbit (Jackson ImmunoResearch Laboratories). The DNA was counterstained with 0.4 µg/ml 4′, 6′-diamidino-2-phenylindole (DAPI) (33258; Sigma) (data not shown). Slides were mounted in 50% glycerol and analyzed within 24 h. As control, the primary antibodies were omitted (data not shown).

### Confocal Scanning Laser Microscopy

All experiments were performed at least two times in duplicate. For each experiment, 100 nuclei were visualized and at least 10 nuclei were imaged. Images were recorded by using a Zeiss LSM 510 Meta confocal laser scanning microscope equipped with a 60X/1.23 NA oil immersion objective. Ar laser (488 and 514 nm), and HeNe laser 1 (543 nm) were used to excite the fluorophores. Emitted fluorescence was detected with a 505- to 530-nm band-pass filter for the green signal and a 560-nm long-pass filter for the red signal. The LSM 510 R. 3.2 META (Zeiss) image analysis software is used.

### ChIPs and Re-ChIPs

In ChIP experiments with animals, 12 weeks old wild type SDF1 and wt C57BL/10 and mdx mice were used. Adductor muscles were isolated and incubated in 1.8% formaldehyde for 20 minutes at 22°C. After 5 minutes muscles were cut in small pieces. The reaction was stopped by addition of glycine to a final concentration of 0,125 M. Cells were incubated in 1% of formaldehyde for 10 minutes at 22°C. The reaction was stopped by addition of glycine to a final concentration of 0,125 M. From this step on ChIP from cells and animal tissues have been performed as previously described [45]. Sonicated chromatin was incubated with 10 µg of anti-PAN-H4ac (Upstate n°06-598), -H3ac-k9 (Abcam n°4441), -H3tri-m-k9 (Abcam n°8898), -H4tri-m-k20 (Abcam n°9053), -HP1α (Abcam n°9057), 4 ug of anti-NF-YA (Rockland n°200-401-100) and -p300 (Santa Cruz n°584 and 585), 4 ul of anti-HDAC1 (Sigma H3284) and 3 ug of anti-NF-YB (gift from R. Mantovani) antibodies. In Re-ChIP experiments, complexes were eluted by incubation for 30 minutes at 37°C in 25 µl 10 mM DTT. After centrifugation, the supernatant was diluted 20 times with re-ChIP buffer (1% Triton X-100, 2 mM EDTA, 150 mM NaCl, 20 mM Tris-HCl, [pH 8.1]) and subjected again to the ChIP procedure. For PCR and Q-PCR analysis 2 ul of template in 20–30 ul of total reaction were used. PCR was performed with HOT-MASTER Taq (Eppendorf). The primers sequences of the mouse promoters were:

mCyclinA2 F5′ CTGTAAGATTCCCGTCGGGCCTTCG,
mCyclinA2 R5′ GTAGAGCCCAGGAGCCGCGAG;
mCyclinB2 F5′ TGTAGACAAGGAAACAACAAAGCCTGGTGGCC,
mCyclinB2 R5′CAGCCACTCCGGTCTGCGACA;
mCyclinB1 F5′AGTCCAACGGAGTCGCCTGG,
mCyclinB1 R5′GAGGAGTCTGCCGGGCTTAG;
mCdk1 F5′TCAAGAGTCAGTTGG CGCCC,
mCdk1 R5′ CACACCGCAGTTCCGGACTG;
mCdc25C F5′AGTCAACGTTAGCGGGGCTC,
mCdc25C R5′ GCCAACAGCCAGAGGTCTGA;
mTopoIIα F5′TCCATTTTGAAGATTCTCCCGCCT,
mTopoIIα R5′ CGAGAATCCGGAAAGCGACAAAAC;
mDhfr F5′CGGCAATCCTAGGGTGAAGGCTGGT,
mDhfr R5′GGCTCCATTCAGCGACGAAAGGTGC;
mTk F5′ CAAGGACCCCCAGCCTTGCACCTT,
mTk R5′AGTTAGCACCGAGTCCGTGGGTGG;
LUC R5′ GGCGTCTTCCATTTTACCAACAGTACCGG;
MLC F5′ CTTCAGTCTCACCAGGGCTGTTCAC,
MLC R5′ CTCTCTCTGGCTTCCTTTTATTTCTTGGGC;
MyoD F5′ TAGACACTGGAGAGGCTTGGGCAG,
MyoD R5′ CCTGGGCTATTTATCCAGGGTAGCC;
Myogenin F5′AAAGGAGAGGGAAGGGGAATCA,
Myogenin R5′GGAAAGGCTTGTTCCTGCCACTGG.
hCycB2 For 5′- CAGAGGCGTCCTACGTCTGC
hCycB2 Rev 5′-TGCGCACGGGTCGCTGTTCT
hCdk1 For 5′- GAACTGTGCCAATGCTGGGA
hCdk1 Rev 5′-GCAGTTTCAAACTCACCGCG
hCdc25C For 5′- GAATGGACATCACTAGTAAGGCGCG
hCdc25C Rev 5′-GCAGGCGTTGACCATTCAAACCTTC

### Q-PCR

Quantitative PCR were performed using SYBR Green (Applied Biosystems) as marker for DNA amplification, on an ABI Prism 7500 apparatus (Applied Biosystems), with 50 cycles of two-step amplification. Each PCR reaction generates only the expected specific amplicons, as assessed by melting-temperature profile and by gel electrophoresis. For every promoter fragment analyzed, each sample was quantified in triplicate from 2 to 5 independent immunoprecipitations. On Q-PCR from animals are represented the mean of five independent experiments performed on triplicate, on Q-PCR from cells is shown one representative experiment done on triplicate. The relative proportions of immunoprecipitated promoter fragments were determined based on the comparative threshold (ΔCt) method (A) and relative quantification method (B) with the same results. On the method B, a standard curve was constructed with serial dilution of the input sample. This curve was then used as a reference standard for extrapolating quantitative information for ChIP sample. The equation used to calculate the relative enrichment of the sample and no-antibody or rabbit IgG (control) relative to input is:
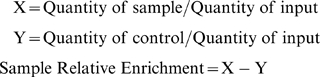



On the method A [17] the ΔCt value was calculated for each sample (x) and for noAb or Rabbit IgG (b) by subtracting the Ct value for the immunoprecipitated sample from the Ct value for the input (i). Relative enrichment was then determined as follow:
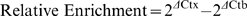
Where:
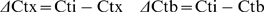



The primers sequences of the mouse promoters used in the PCR reactions are:

mcdc25C F5′GATGATTGGCTGACGCTGCT,
mcdc25C R5′ CCTGAAGGCAAAGCCAACAG;
mCyclinA2 F5′TCCCGCCCTGTAAGATTCC,
mCyclinA2 R5′ CAAGTAGCCCGCACTATTGA;
mCyclinB2 F5′ TGAAAATAAGACTGTAGACAAGGAAACAAC,
mCyclinB2 R5′ GCTGGCTTGTTTGCTTGTCATA;
mCyclinB1 F5′ GAGTCGCCTGGGCTAGCTT,
mCyclinB1 R5′ CTGCGCACAAGTCGCATT;
mDhfr F5′GATTTCGCGCCAAACTTGAC,
mDhfr R5′ CGACGATGCAGTTCAATGGT;
mTopo2α F5′AGAAGGACGGCTTAGTCAACCA,
mTopo2α R5′CCAATGAGGAAGGTCCATGTG;
mCdk1 F5′CAGCTCTGATTGGCTCCTTTG,
mCdk1 R5′CCGAGTGCCAGCAGTTTCA;
mCyclinA2 F5′ CGCCCTCATTGGTTTATTTCA,
mCyclinA2 R5′ GAACTACAAGACCAGCAGCCG;
mTk F5′CCTGCCTTACTTTGCGACTCA,
mTk R5′GGTTGTGCAGTGATTCTGTGT;
MLC F5′ CTTCAGTCTCACCAGGGCTGTTCAC,
MLC R5′ CTCTCTCTGGCTTCCTTTTATTTCTTGGGC;
Myogenin F5′ TCACATGTAATCCACTGGAAACGT,
Myogenin R5′ AAACCTGAGCCCCCCTCTAA.

### RNA extraction and RT-PCR

Total RNA was extracted using the Trizol Reagent (Gibco BRL) and following the manufacturer's instructions. The first strand of cDNA was synthetized according to manufacture instructions of the M-MLV RT kit (Invitrogen). PCR was performed with HOT-MASTER Taq (Eppendorf) using 2 µl of cDNA reaction. The primers sequences of the mouse genes are:

RT-mCdk1 F5′GGGAATTGTGTTTTGCCACT,
RT-mCdk1 R5′GATGTCAACCGGAGTGGAGT;
RT-mCdc25c F5′CTGAGGAAGCCTGTTGTTCC,
RT-mCdc25c R5′AATGCCGGATACTGGTTCAG;
RT-mCyclinA2 F5′CTTGGCTGCACCAACAGTAA,
RT-mCyclinA2 R5′AGCAATGAGTGAAGGCAGGT;
RT-mTK F5′GATGGGACCTTCCAGAGGA,
RT-mTK R5′CCTCTTCGTGTAGGCAGCTT;
RT-mTopo2α F5′AGGACAGTGTGCCAGCTTCT,
RT-mTopo2α R5′ACGAGCACTCAAGGCTGAAT;
RT-mAld F5′-TGG ATG GGC TGT CTG AAC GCT GT,
RT-mCycB2 F5′CTCTGCTCCTGGCTTCCAAA,
RT-mCycB2 R5′GTTCAACATCCACCTCTCC;
RT-mAld R5′-AGT GAC AGC AGG GGG CAC TGT;
RT-Armcx2 F5′ GAACTGTCTTTATCCGTCTAC,
RT-Armcx2 R5′ CTGGGATCAGGAACA ATT TC.

### Immunohistochemistry

Immunohistochemical staining was carried out on 5 µm thick sections cut on SuperFrost®Plus slides (Menzel-Glaser, Menzel GmbH & Co) from routinely formalin fixed paraffin embedded blocks. Adductor sections from wt and MDX C57BL/10 mice, pretreated in a thermostatic bath at 96°c for 40 minutes in 10 mM citrate buffer (pH6.0), were processed by using l anti-NF-YA rabbit polyclonal antibody (Rockland) and a streptavidin-biotin immunoperoxidase system (Dako). Peroxidase activity of the secondary antibodies was visualized by reaction with 3,3′-Diaminobenzidine (DAB) (Dako) and H_2_O_2_ in sodium phosphate buffer.

## References

[1] Ehrenhofer-Murray AE (2004). Chromatin dynamics at DNA replication, transcription and repair.. Eur J Biochem.

[2] Fischle W, Wang Y, Allis CD (2003). Histone and chromatin cross-talk.. Curr Opin Cell Biol.

[3] Li B, Carey M, Workman JL (2007). The Role of Chromatin during Transcription.. Cell.

[4] Strahl BD, Allis CD (2000). The language of covalent histone modifications.. Nature.

[5] Barski A, Cuddapah S, Cui K, Roh TY, Schones DE (2007). High-resolution profiling of histone methylations in the human genome.. Cell.

[6] Berger SL (2007). The complex language of chromatin regulation during transcription.. Nature.

[7] Miao F, Natarajan R (2005). Mapping Global Histone Methylation Patterns in the Coding Regions of Human Genes.. Mol Cell Biol.

[8] Peterson CL, Laniel MA (2004). Histones and histone modifications.. Curr Biol.

[9] Agger K, Cloos PA, Christensen J, Pasini D, Rose S (2007). UTX and JMJD3 are histone H3K27 demethylases involved in HOX gene regulation and development.. Nature..

[10] Ait-Si-Ali S, Guasconi V, Fritsch L, Yahi H, Sekhri R (2004). A Suv39h-dependent mechanism for silencing S-phase genes in differentiating but not in cycling cells.. The EMBO Journal.

[11] Meshorer E, Misteli T (2006). Chromatin in pluripotent embryonic stem cells and differentiation.. Nat Rev Mol Cell Biol.

[12] Schlesinger Y, Straussman R, Keshet I, Farkash S, Hecht M (2007). Polycomb-mediated methylation on Lys27 of histone H3 pre-marks genes for de novo methylation in cancer.. Nat Genet.

[13] Brehm A, Kouzarides T (2000). Regulation of E2F1 activity by acetylation.. EMBO J.

[14] Glozak MA, Sengupta N, Zhang X, Seto E (2005). Acetylation and deacetylation of non-histone proteins.. Gene.

[15] Amati B, Frank SR, Donjerkovic D, Taubert S (2001). Function of the c-Myc oncoprotein in chromatin remodeling and transcription.. Biochim Biophys Acta.

[16] Di Agostino S, Strano S, Emiliozzi V, Zerbini V, Mottolese M (2006). Gain of function of mutant p53: the mutant p53/NF-Y protein complex reveals an aberrant transcriptional mechanism of cell cycle regulation.. Cancer Cell.

[17] Frank SR, Schroeder M, Fernandez P, Taubert S, Amati B (2001). Binding of c-Myc to chromatin mediates mitogen-induced acetylation of histone H4 and gene activation.. Genes & Development.

[18] Guccione E, Martinato F, Finocchiaro G, Luzi L, Tizzoni L (2006). Myc-binding-site recognition in the human genome is determined by chromatin context. Nat.. Cell Biol.

[19] Nielsen SJ, Schneider R, Bauer UM, Bannister AJ, Morrison A (2001). Rb targets histone H3 methylation and HP1 to promoters.. Nature.

[20] Taubert S, Gorrini C, Frank SR, Parisi T, Fuchs M (2004). E2F-dependent histone acetylation and recruitment of the Tip60 acetyltransferase complex to chromatin in late G1. Mol Cell Biol..

[21] Caretti G, Motta MC, Mantovani R (1999). NF-Y associates H3-H4 tetramers and octamers by multiple mechanisms.. Mol Cell Biol.

[22] Mantovani R (1998). A survey of 178 NF-Y binding CCAAT boxes.. Nucleic Acids Res.

[23] Romier C, Cocchiarella F, Mantovani R, Moras D (2003). The NF-YB/NF-YC structure gives insight into DNA binding and transcription regulation by CCAAT factor NF-Y.. J Biol Chem.

[24] Bolognese F, Wasner M, Dohna CL, Gurtner A, Ronchi A (1999). The cyclin B2 promoter depends on NF-Y, a trimer whose CCAAT-binding activity is cell-cycle regulated.. Oncogene.

[25] Farina A, Manni I, Fontemaggi G, Tiainen M, Cenciarelli C (1999). Down-regulation of cyclin B1 gene transcription in terminally differentiated skeletal muscle cells is associated with loss of functional CCAAT-binding NF-Y complex.. Oncogene.

[26] Hu Q, Maity SN (2000). Stable expression of a dominant negative mutant of CCAAT binding factor/NF-Y in mouse fibroblast cells resulting in retardation of cell growth and inhibition of transcription of various cellular genes.. J Biol Chem.

[27] Imbriano C, Gurtner A, Cocchiarella F, Di Agostino S, Basile V (2005). Direct p53 transcriptional repression: *in vivo* analysis of CCAAT-containing G2/M promoters.. Mol Cell Biol.

[28] Manni I, Mazzaro G, Gurtner A, Mantovani R, Haugwitz U (2001). NF-Y mediates the transcriptional inhibition of the cyclin B1, cyclin B2, and cdc25C promoters upon induced G2 arrest.. J Biol Chem.

[29] Matsui T, Katsuno Y, Inoue T, Fujita F, Joh T (2004). Negative regulation of Chk2 expression by p53 is dependent on the CCAAT-binding transcription factor NF-Y.. J Biol Chem.

[30] Matuoka K, Yu Chen K (1999). Nuclear factor Y (NF-Y) and cellular senescence.. Exp Cell Res.

[31] Okamura H, Yoshida K, Sasaki E, Morimoto H, Haneji T (2004). Transcription factor NF-Y regulates mdr1 expression through binding to inverted CCAAT sequence in drug-resistant human squamous carcinoma cells.. Int J Oncol.

[32] Ru Lee W, Chen CC, Liu S, Safe S (2006). 17beta-estradiol (E2) induces cdc25A gene expression in breast cancer cells by genomic and non-genomic pathways.. J Cell Biochem.

[33] Sciortino S, Gurtner A, Day A, Sacchi A, Ozato K (2001). The cyclin B1 gene is actively transcribed during mitosis in HeLa cells.. EMBO Rep.

[34] Van Ginkel PR, Hsiao KM, Schjerven H, Farnham PJ (1997). E2F-mediated growth regulation requires transcription factor cooperation.. J Biol Chem.

[35] Elkon R, Linhart C, Sharan R, Shamir R, Shiloh Y (2003). Genome-wide in silico identification of transcriptional regulators controlling the cell cycle in human cells.. Genome Res.

[36] Linhart C, Elkon R, Shiloh Y, Shamir R (2005). Deciphering transcriptional regulatory elements that encode specific cell cycle phasing by comparative genomics analysis.. Cell Cycle.

[37] Grskovic M, Chaivorapol C, Gaspar-Maia A, Li H, Ramalho-Santos M (2007). Systematic identification of cis-regulatory sequences active in mouse and human embryonic stem cells.. PLoS Genet.

[38] Bhattacharya A, Deng JM, Zhang Z, Behringer R, de Crombrugghe B (2003). The B Subunit of the CCAAT Box Binding Transcription Factor Complex (CBF/NF-Y) Is Essential for Early Mouse Development and Cell Proliferation.. Cancer Research.

[39] Currie RA (1998). NF-Y is associated with the histone acetyltransferases GCN5 and P/CAF.. J Biol Chem.

[40] Jin S, Scotto KW (1998). Transcriptional regulation of the MDR1 gene by histone acetyltransferase and deacetylase is mediated by NF-Y.. Mol Cell Biol.

[41] Li Q, Herrler M, Landsberger N, Kaludov N, Ogryzko VV (1998). Xenopus NF-Y pre-sets chromatin to potentiate p300 and acetylation-responsive transcription from the Xenopus hsp70 promoter *in vivo*.. EMBO J.

[42] Peng Y, Stewart D, Li W, Hawkins M, Kulak S (2007). Irradiation modulates association of NF-Y with Histone-modifying cofactors PCAF and HDAC.. Oncogene.

[43] Salsi S, Caretti G, Wasner M, Reinhard W, Haugwitz U (2003). Interactions between p300 and multiple NF-Y trimers govern cyclin B2 promoter function.. J Biol Chem.

[44] Caretti G, Salsi V, Vecchi C, Imbriano C, Mantovani R (2003). Dynamic recruitment of NF-Y and histone acetyltransferases on cell-cycle promoters.. J Biol Chem.

[45] Gurtner A, Manni I, Fuschi P, Mantovani R, Guadagni F (2003). Requirement for down-regulation of the CCAAT-binding activity of the NF-Y transcription factor during skeletal muscle differentiation.. Mol Biol Cell.

[46] Wansink DG, Schul W, Van der Kraan I, Van Steensel B, Van Driel R (1993). Fluorescent Labeling of Nascent RNA Reveals Transcription by RNA Polymerase II in Domains Scattered Throughout the Nucleus.. J Cell Biol.

[47] Cho Eun-Jung (2007). RNA polymerase II carboxy-terminal domain with multiple connections.. Exp Mol Med.

[48] Lachner M, Jenuwein T (2002). The many faces of histone lysine methylation.. Curr Opin Cell Biol.

[49] Bannister AJ, Zegerman P, Partridge JF, Miska EA, Thomas JO (2001). Selective recognition of methylated lysine 9 on histone H3 by the HP1 chromodomain.. Nature.

[50] Rando TA, Blau HM (1994). Primary mouse myoblast purification, characterization, and transplantation for cell-mediated gene therapy.. J Cell Biol.

[51] Park S-H, Yu G–R, Kim W–H, Moon W–S, Kim J–H (2007). NF-Y-Dependent Cyclin B2 Expression in Colorectal Adenocarcinoma.. Clin Cancer Res.

[52] Mantovani R, Li XY, Pessara U, Hooft van Huijsduijnen R, Benoist C (1994). Dominant negative analogs of NF-YA.. J Biol Chem.

[53] Wienche JK, Zheng S, Morrison Z, Yeh R-F (2007). Differentially expressed genes are marked by histone 3 lysine 9 trimethylation in human cancer cells.. Oncogene. Oct 29; [Epub ahead of print].

[54] Bulfield G, Siller WG, Wight PA, Moore KJ (1984). X chromosome-linked muscular dystrophy (*mdx*) in the mouse.. Proc Natl Acad Sci USA.

[55] de Wit E, Greil F, van Steensel B (2007). High-resolution mapping reveals links of HP1 with active and inactive chromatin components.. PLoS Genet.

[pone.0002047-Huang1] Huang W, Zhao S, Ammanamanchi S, Brattain M, Venkatasubbarao K (2005). Trichostatin A induces transforming growth factor beta type II receptor promoter activity and acetylation of Sp1 by recruitment of PCAF/p300 to a Sp1.NF-Y complex.. J Biol Chem.

[57] Bernstein BE, Mikkelsen TS, Xie X, Kamal M, Huebert DJ (2006). A bivalent chromatin structure marks key developmental genes in embryonic stem cells.. Cell.

[58] Spivakov M, Fisher AG (2007). Epigenetic signatures of stem-cell identity.. Nat Rev Genet.

[59] Barsyte-Lovejoy D, Lau SK, Boutros PC, Khosravi F, Jurisica I (2006). The c-Myc oncogene directly induces the H19 noncoding RNA by allele-specific binding to potentiate tumorigenesis.. Cancer Res.

[60] Knoepfler PS, Zhang XY, Cheng PF, Gafken PR, McMahon SB (2005). Myc influences global chromatin structure.. EMBO J.

[61] Haukenes G, Kalland K-H (1998). Labelling of Eukaryotic RNA Transcripts With BrUTP Using a Non-Liposomal Tranfection Reagent (FuGENE(TM)6): New Application for Transfection Reagent.. Biochemica.

